# Clinical potential of inclisiran for patients with a high risk of atherosclerotic cardiovascular disease

**DOI:** 10.1186/s12933-023-01752-4

**Published:** 2023-01-30

**Authors:** Toshiyuki Nishikido

**Affiliations:** 1 Department of Cardiovascular Medicine, National Hospital Organization Kobe Medical Center, Nishiochiai 3-1-1, Suma-ku, Kobe City, Japan; 2 Department of Cardiovascular Medicine, Saga University, Nabeshima 5-1-1, Saga City, Japan

**Keywords:** Inclisiran, Lipid-lowering therapies, Low-density lipoprotein cholesterol, Proprotein convertase subtilisin/kexin type 9, Small interfering ribonucleic acid, Individual variability, Treatment adherence

## Abstract

Elevated low-density lipoprotein cholesterol (LDL-C) level is associated with an increased risk of atherosclerotic cardiovascular disease. Although high-intensity lipid-lowering therapies with statins and ezetimibe are highly effective for reducing LDL-C levels, over half of high-risk patients do not achieve guideline-recommended LDL-C goals. Thus, there is a significant gap between treatment guidelines and their implementation in daily clinical practice. The major causes are individual variability in the response to lipid-lowering therapies and variation in treatment adherence. Proprotein convertase subtilisin/kexin type 9 (PCSK9) monoclonal antibodies combined with statins provide marked and consistent reduction in LDL-C levels; however, poor adherence due to the need for subcutaneous injections every 2 or 4 weeks and high cost are major obstacles to their use in real-world clinical settings. Inclisiran, a recently approved novel small interfering ribonucleic acid (siRNA) molecule that inhibits PCSK9 synthesis, provides robust and long-term reduction in LDL-C levels with a low inter-individual variability in the LDL-C-lowering response. Moreover, its administration by biannual injection is expected to greatly improve treatment adherence. Clinical trials of this drug lasting for up to 4 years showed acceptable safety profiles, and ongoing studies accumulate evidence of its longer-term safety. This narrative review summarizes the available evidence on the efficacy and safety of inclisiran and analyzes its potential to overcome the gap between guideline recommendations and real-world clinical practice in current LDL-C-lowering therapies, with a focus on reduced LDL-C level variability and improved treatment adherence.

## Introduction

Elevated low-density lipoprotein cholesterol (LDL-C) level is a causal factor for developing atherosclerotic cardiovascular disease (ASCVD), and evidences from randomized clinical trials, cohort studies, and Mendelian randomized studies have demonstrated a consistent dose-dependent log-linear association between absolute magnitude of exposure to LDL-C and the risk of ASCVD [1, 2]. Conversely, absolute reduction in LDL-C levels is associated with a proportional reduction in the risk of ASCVD, regardless of the presence of other risk factors. A meta-analysis of data from 26 randomized trials involving intensive statin therapy revealed that there was a 22% relative reduction in the rate of major cardiovascular events per 1 mmol/L (38.7 mg/dL) reduction in LDL-C levels [3]. Furthermore, achieving very low levels of LDL-C has beneficial effects on the risk for major cardiovascular events [4]. In a post-hoc analysis of the JUPITER trial, patients who achieved LDL-C levels below 50 mg/dL with high-intensity statin therapy experienced the fewest cardiovascular events without an increase in the rate of systemic adverse events [5]. Additionally, the IMPROVE-IT trial showed that adding ezetimibe to statin therapy provided incremental reduction in LDL-C levels by 53.2 mg/dL and improved cardiovascular outcomes for secondary prevention [6, 7]. In particular, high-risk patients with multiple commodities who are likely to have a high cardiovascular event rate despite statin therapy had an increased relative and absolute benefit from the addition of ezetimibe [8].

Therefore, the intensive LDL-C-lowering strategy was widely accepted and current guidelines for the management of dyslipidemia recommend more aggressive LDL-C reduction to achieve the target level for high-risk populations. For secondary prevention, the 2019 European Society of Cardiology/European Atherosclerosis Society Guideline for the Management of Dyslipidemias recommends ≥ 50% reduction from baseline values and an LDL-C target level of < 55 mg/dL, whereas the 2018 American College of Cardiology/American Heart Association Multisociety Guideline on the Management of Blood Cholesterol uses an LDL-C threshold of 70 mg/dL for initiation of non-statin therapy on top of statin therapy [9, 10]. However, many high-risk patients receiving statins fail to achieve the guideline-recommended reduction in LDL-C levels in real-world clinical setting. The DAVINCI study and EUROASPIRE V survey across European countries revealed that only half of the patients with established ASCVD received high-intensity statin therapy, and less than half achieved the target LDL-C level of < 70 mg/dL in secondary prevention [11, 12].

Inclisiran is a long-acting, small interfering ribonucleic acid (siRNA) that lowers consistently circulating LDL-C levels by approximately 50% with a twice-yearly dosing regimen [13]. Prior reviews of inclisiran therapy have not focused on its potential to reduce the gap between current guideline recommendations and their implementation. Therefore, this narrative review briefly summarizes the development of therapeutic agents with a potential to achieve this goal and focuses on the role of inclisiran in real-world clinical settings, with an overview of its pharmacological properties and the current evidence supporting its potential to provide robust and long-term reduction in LDL-C levels with low interindividual variability and improve treatment adherence for high-risk populations who have established ASCVD or ASCVD-risk equivalents with elevated LDL-C levels.

## Novel strategies to target proprotein convertase subtilisin/kexin type 9

The LDL receptors (LDLRs) on the surface of hepatocytes control plasma LDL-C levels by binding to circulating LDL-C and subsequent internalization of the LDL-C/LDLR complex via clathrin-mediated endocytosis. In the acidic compartment of endosomes, LDL-C is released from the LDLR and degraded in lysosomes, while the LDLR is recycled to the surface of hepatocytes. LDLR expression is regulated at the transcriptional level by a negative feedback mechanism in the intracellular cholesterol pool [14, 15].

**Fig. 1:**
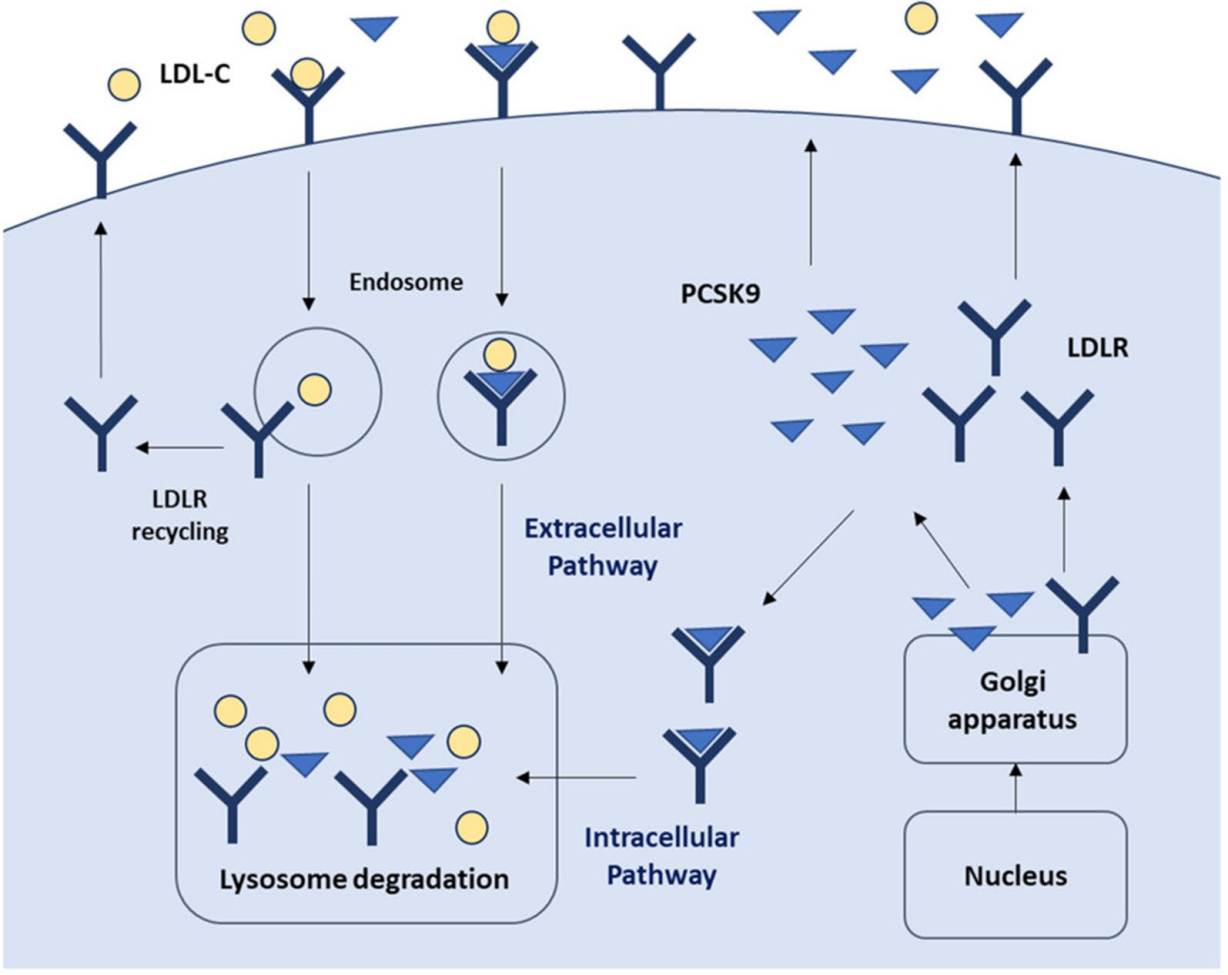
The role of PCSK9 in lipid metabolism. LDLRs on the surface of the liver cells bind circulating LDL, and LDL/LDLR complexes are internalized via clathrin‐mediated endocytosis. LDL is released for degradation in the lysosome, while the LDLR is recycled to the cell surface. In extracellular pathway, PCSK9 binds to the LDLR on the surface of the liver cell and then internalizes with LDLR to intracellular degradation in the lysosome, which increase serum LDL levels by preventing LDLR recycling to the membrane. In intracellular pathway, the secreted PCSK9 from Golgi apparatus can be sorted directly to lysosomes as a PCSK9–LDLR complex, leading to intracellular degradation of the LDLR. *LDLR* LDL receptors, *PCSK9* proprotein convertase subtilisin/kexin type 9

Proprotein convertase subtilisin/kexin type 9 (PCSK9), expressed predominantly in the liver and, to a lesser extent, in the small intestine, kidneys, pancreas, and the central nervous system, plays an important role in the regulation of LDL-C homeostasis via two pathways [16]. In the extracellular pathway, circulating PCSK9 binds to LDLRs on the hepatocyte surface and forms the PCSK9/LDLR complex, which is internalized via clathrin-mediated endocytosis and then directed to the lysosome for degradation of LDLRs. This degradation results in low levels of LDLRs at the cellular membrane of hepatocytes, leading to reduction in LDL-C uptake and increase in plasma LDL-C levels. In the intracellular pathway, PCSK9 enhances intracellular LDLR degradation by direct intracellular trafficking of the PCSK9/LDLR complex from the trans-Golgi network to the lysosomes without recycling LDLRs to the cellular surface [17–19] (Fig. 1).

The *PCSK9* gene was identified as a third causative gene for familial hypercholesterolemia [20]. Gain-of-function mutations in this gene promote degradation of LDLRs, resulting in higher LDL-C levels and increased ASCVD risk. In contrast, loss-of-function mutations in the *PCSK9* gene were found to be associated with reduced LDLR degradation, leading to increased LDLR expression, lower LDL-C levels, and substantial reduction in the rate of cardiovascular events [21, 22]. In addition, individuals with severe loss-of-function mutations in the *PCSK9* gene were apparently healthy without physical and neurological impairment despite the very low LDL-C levels (< 14 mg/dL) from birth [23]. These findings suggested that PCSK9 would be a promising therapeutic target for LDL-C level reduction for ASCVD prevention.

## Anti-PCSK9 monoclonal antibodies

As a first approach to inhibit PCSK9, monoclonal antibodies (mAbs) were used to selectively prevent PCSK9 binding to LDLRs. Anti-PCSK9 mAbs bind to the catalytic domain of plasma extracellular PCSK9 with high affinity and specificity, blocking the interaction between LDLRs and PCSK9. Thus, PCSK9-mediated reduction in LDLR expression is attenuated, resulting in increased LDL-C uptake and subsequent reduction in plasma LDL-C levels [24]. Two fully human anti‐PCSK9 mAbs, alirocumab (SAR236553/REGN727) and evolocumab (AMG145), were eventually approved for clinical use by the United States Food and Drug Administration and the European Medicines Agency.

Subcutaneous injection of anti-PCSK9 mAbs every 2 or 4 weeks provides remarkable reduction in LDL-C levels by up to approximately 60% without serious adverse events in patients with maximally tolerated statin therapy. More importantly, two large randomized controlled trials, the FOURIER and ODYSSEY Outcomes trials, demonstrated not only a 50–60% reduction in LDL-C levels but also cardiovascular event risk reduction of approximately 15–20% in patients receiving statin therapy for secondary prevention [25–27]. The 2019 European Society of Cardiology/European Atherosclerosis Society guideline, 2018 American College of Cardiology/American Heart Association Multi-society guideline, and 2022 American College of Cardiology Expert Consensus for non-statin therapies acknowledge PCSK9 inhibitors as valuable options for combined LDL-C-lowering therapy in patients with very high cardiovascular risk [9, 10, 28].

Nevertheless, there are still several barriers to the wide implementation of anti-PCSK9 mAbs in daily clinical practice, such as the complex administration regimen, poor adherence, high cost, limited accessibility, reimbursement limitation, and lack of long-term clinical safety data.

## Inclisiran: a small interfering ribonucleic acid-based inhibitor of PCSK9

### Molecular structure and mechanism of action

Inclisiran (ALN-PCSSC; ALN-60212) is a first-in-class siRNA that inhibits the hepatic synthesis of PCSK9 by RNA interference, resulting in significant and long-term reduction in LDL-C levels. This duplex RNA contains one 2′-deoxy, 11 2′-fluoro, and 32 2′-*O*-methyl modified nucleotides. It consists of two complementary RNA strands, an antisense (guide) strand, and a sense (passenger) strand. The 3′ end of the sense strand is conjugated to the synthetic ligand triantennary *N*-acetyl galactosamine (GalNAc), which is a ligand of asialoglycoprotein receptors (ASGPRs), expressed mainly on the surface of hepatocytes. The siRNA enters the hepatocytes through the interaction between GalNAc and ASGPRs, which provides precise and rapid hepatic uptake. In addition, the formulated lipid nanoparticle encapsulation helps to increase endocytosis into target cells despite the high molecular weight and negative electrical charge of the siRNA [29].

**Fig. 2:**
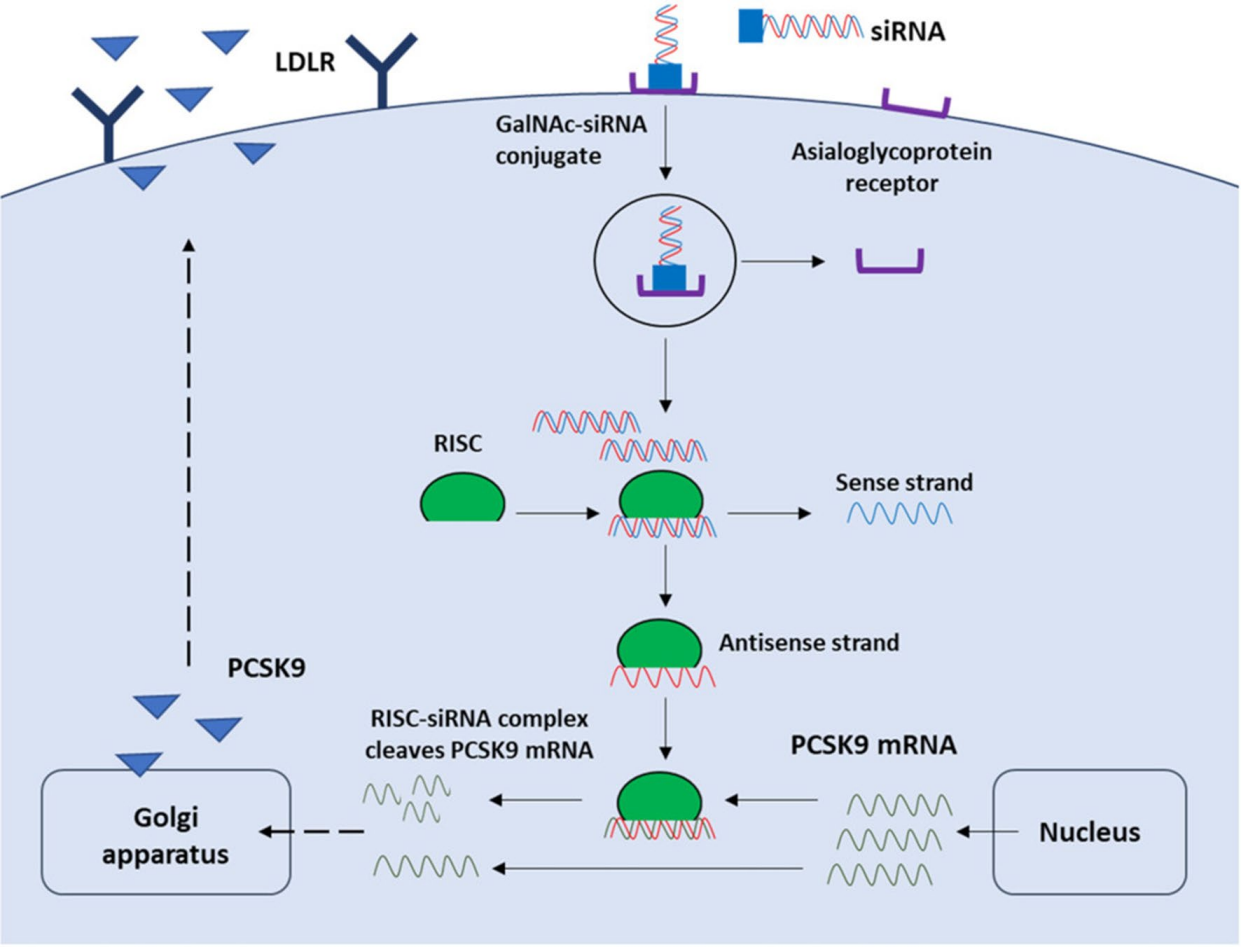
The mechanism of PCSK9 synthesis inhibition by inclisiran. Inclisiran, GalNAc‐conjugated siRNA, binds to the asialoglycoprotein receptor on the surface of hepatocytes with high selectivity, which can be delivered into the cell via endocytosis. The antisense strand of the siRNA duplex is incorporated into the RNA-induced silencing complex intracellularly, which cleaves mRNA encoding PCSK9 specifically. The inhibition of PCSK9 synthesis reduces the degradation of LDL receptors. *RISC* ribonucleic acids‐induced silencing complex, *ASGPRs* asialoglycoprotein receptors, *GalNAc* *N*-acetyl galactosamine

When the siRNA is loaded into the RNA induced-silencing complex (RISC) in hepatocytes, only the antisense strand is activated via selective removal of the sense strand by the Argonaute 2 [30]. This complex of the antisense strand and RISC binds to PCSK9 messenger RNA (mRNA) transcripts, and selectively and catalytically cleaves mRNA, inhibiting the translation of complementary mRNA transcript [31]. The consequent reduced synthesis of the PCSK9 protein results in significantly reduced plasma LDL-C levels (Fig. 2). As only approximately 100–200 loaded RISC complexes per cell are sufficient to eliminate the gene expression, each complex has a very long half-life from multiple mRNA copies, allowing dosing in patients to be months apart. In addition, the GalNAc-mediated liver specificity allows for use of lower cumulative doses, enabling subcutaneous administration [32].

These mechanisms give inclisiran the advantage of safety and less frequent administration. Subcutaneous administration at 0 and 3 months and every 6 months thereafter can provide significant reduction in LDL-C levels by approximately 50% in high-risk and very high-risk patients, as well as patients with statin intolerance.

### Pharmacokinetics and pharmacodynamics

ALN-PCS, the early predecessor of inclisiran formulated in a lipid nanoparticle, reduced PCSK9 mRNA levels by 70% and LDL-C levels by 60% in animal models with a duration of 3 weeks [29]. In healthy volunteers who received ALN-PCS by intravenous infusion for 60 min, the highest dose of ALN-PCS reduced PCSK9 mRNA and protein levels by 70% and LDL-C levels by 40% after 3 days, and the effect sustained for 2 to 3 weeks after administration [33]. ALN-PCSsc (inclisiran), made by refinement of ALN-PCS, induced the optimal multivalent design of siRNA-GalNAc, enabling better stability and prolonged activity. ALN-PCSsc can be administered by subcutaneous injection. Tissue-specific delivery of ALN-PCSsc led to a potent and dose-dependent inhibition of PCSK9 gene expression. In healthy volunteers administered a single subcutaneous dose of inclisiran (100, 300, 500, or 800 mg), the least-square mean LDL-C reduction from baseline to day 84 was 36.7%, 50.0%, 50.6%, and 43.4%, respectively. Additionally, a dose of 300 mg or higher maintained PCSK9 and LDL-C level reduction over 6 months [34].

Following subcutaneous administration of a single dose in the range of 24–756 mg, exposure to inclisiran appears to increase in approximately dose-proportional manner. Peak plasma concentration was reached within 4 h after administration of the recommended dose (284 mg: equivalent 300 mg inclisiran sodium). It is rapidly cleared from the circulation through hepatic uptake and renal elimination, and is undetectable in the plasma by 48 h after administration. The major route of systemic clearance from the circulation is through hepatic uptake, with 16% being cleared through renal elimination [35]. Once in the tissue, it is mainly metabolized by non-specific nucleases to an inactive shorter nucleotide [36, 37]. The limited systemic exposure due to rapid clearance provides the benefit of lower theoretical risk for off-target effects.

Although individuals with mild or moderate hepatic and renal impairment had greater exposure to inclisiran, the pharmacodynamic effects remained relatively unchanged in terms of LDL-C level reduction, clearance, duration of effects, and safety [38, 39]. However, as the effect of inclisiran has not been evaluated in patients with severe hepatic impairment (Child–Pugh class C), its use in these patients should be cautioned. Inclisiran is not anticipated to have clinically significant interactions with other drugs, although limited data are available.

### Overview of clinical trials

In a phase 1 trial (NCT02314442), healthy volunteers with LDL-C levels higher than 100 mg/dL were subcutaneously administered inclisiran in either a single-dose regimen (25–800 mg) or multiple-dose regimens of 125–500 mg with intervals of at least 1 week. At day 84, the maximal reduction by the single-dose regimen was 74.5% in PSCK9 levels after a 300 mg dose and 50.6% in LDL-C levels after a 500 mg dose. All multiple-dose regimens of inclisiran reduced LDL-C and PCSK9 levels by up to 59.7% and 83.8%, respectively. After administration of doses of 300 mg or higher, the reduction in LDL-C and PCSK9 levels was durable over at least 180 days. There were no serious adverse events versus placebo in this trial [34].

Based on these results, the ORION trials were initiated as a global clinical development program to evaluate the efficacy and safety of inclisiran [40] (Table 1). In phase 1 trials, the ORION-6 and ORION-7 (NCT03159416) trials demonstrated a favorable safety profile of inclisiran in patients with hepatic and renal impairment, respectively [38, 39], while the ORION-12 trial confirmed that there is no clinically significant effect on cardiac repolarization assessed by the QTc interval or other electrocardiographic parameters [41].

**Table 1:** Summary of ORION and VICTORION programs

Trial	Phase	Description	Population	Participants	Country	Status
ORION-1 (NCT02597127)	II	Efficacy and Safety in different doings	ASCVD or risk equivalent	501	Multi-countries	Completed
ORION-2 (NCT02963311)	II	Efficacy and safety in HoFH	HoFH	9	Multi-countries	Completed
ORION-3 (NCT03060577)	II	Open-label extension study of ORION-1	ASCVD or risk equivalent	382	Multi-countries	Completed
ORION-4 (NCT03705234)	III	Cardiovascular outcome study	ASCVD	15,000	UK, US	On going
ORION-5 (NCT03851705)	III	Efficacy and safety in HoFH	HoFH	56	Multi-countries	Completed
ORION-6	I	Hepatic pharmacokinetics	Hepatic impairment	24	US	Completed
ORION-7 (NCT03159416)	I	Renal pharmacokinetics	Renal impairment	31	US	Completed
ORION-8 (NCT03814187)	III	Long term extension study of ORION-3/9/10/11	ASCVD or risk equivalent, FH	3275	Multi-countries	On going
ORION-9 (NCT03397121)	III	Efficacy and safety in HeFH	HeFH	482	Multi-countries	Completed
ORION-10 (NCT03399370)	III	Efficacy and safety in US	ASCVD	1561	US	Completed
ORION-11 (NCT03400800)	III	Efficacy and safety in EU	ASCVD or risk equivalent	1617	Multi-countries	Completed
ORION-12	I	Thorough QT/QTc	Healthy volunteers	48	US	Completed
ORION-13 (NCT04659863)	III	Efficacy and safety in HoFH adolescents	Adolescents with HoFH	12	Multi-countries	On going
ORION-14 (NCT04774003)	I	Efficacy and safety in Chinese	Chinese	40	China	Completed
ORION-15 (NCT04666298)	II	Efficacy and safety in Japanese	Japanese with ASCVD	312	Japan	Completed
ORION-16 (NCT04652726)	III	Efficacy and safety in HeFH adolescents	Adolescents with HeFH	150	Multi-countries	On going
ORION-17	III	Primary prevention trial	–	40,000	UK	In planning
ORION-18 (NCT04765657)	III	Efficacy and safety in Asian	ASCVD or risk equivalent	345	Asia	On going
VICTORION-INCEPTION (NCT04873934)	III	Inclisiran vs. usual care	Recent ACS	384	US	On going
VICTORION-2PREVENT (NCT05030428)	III	MACE	ASCVD	15,000	Multi-countries	On going
VICTORION-INICIATE (NCT04929249)	III	Inclisiran first vs. usual care	ASCVD	444	US	On going
VICTORION-IMPLEMENT (NCT05362903)	–	Inclisiran prospective cohort	Elevated LDL-C	2030	Germany	On going
VICTORION-REAL (NCT05399992)	–	Efficacy and adherence prospective cohort	ASCVD or risk equivalent or HeFH	2100	Switzerland	On going
V-DIFERENCE (NCT05192941)	IV	Efficacy, safety and quality of life	ASCVD or risk equivalent or HeFH	1,760	EU	On going
VICTORION-SPIRIT (NCT04807400)	III	Implementation, preference and utility for administration	ASCVD or risk equivalent	898	UK	On going

*ASCVD* atherosclerotic cardiovascular disease, *LDL-C* low-density lipoprotein cholesterol, *MACE* major adverse cardiovascular events, *ACS* acute coronary syndrome, *HeFH* heterozygous familial hypercholesterolemia, *HoFH* homozygous familial hypercholesterolemia

The ORION-1 trial (NCT02597127), the first phase 2 trial, was a multicenter, double-blind, placebo-controlled trial that enrolled 501 patients at high risk of ASCVD with elevated LDL-C levels [42]. Approximately 73% of the enrolled patients were receiving statin therapy. In this dose-finding study, patients received a single dose of inclisiran of 200, 300, or 500 mg, or placebo, or two doses of inclisiran of 100, 200, or 300 mg, or placebo (days 1 and 90). The primary endpoint was the change in LDL-C levels on day 180. At day 30 after the first injection, the PCSK9 level was reduced by 66.2–74.0% and the LDL-C level was reduced by 44.5–50.5%, depending on the received dose. At day 180, the least square mean reduction in LDL-C levels from baseline was 27.9–41.9% after a single dose and 35.5–52.6% after two doses. The greatest LDL-C level reduction was observed after two doses of inclisiran of 300 mg. Additionally, the mean reduction in LDL-C levels after 240 days was 26.7–47.2%, and the reduction in PCSK9 and LDL-C levels remained consistent across all dose regimens at day 240. These findings suggested that biannual dosing was appropriate as the most efficient administration regimen for inclisiran. Moreover, the prespecified analysis revealed that inclisiran provided sustained and concordant reduction in apolipoprotein B and non-HDL-C levels over 210 days, as well as modest reductions in VLDL-C and triglyceride levels [43]. The incidence of adverse events was similar in the inclisiran and placebo groups, with serious adverse event rates of 11% and 8%, respectively. Injection-site reactions occurred in 4% of patients who received a single dose and in 7% of those who received two doses of inclisiran.

The ORION-3 trial (NCT03060577), a phase 2 open-label extension of the ORION-1 trial, assessed the long-term efficacy and safety of inclisiran for up to 4 years in patients who previously received inclisiran in the ORION-1 trial and patients who received evolocumab for 1 year [44]. The median duration of exposure to inclisiran from baseline through ORION-3 was 4.5 years. The patients who received biannual injection of inclisiran achieved LDL-C level reduction of 47.5% from baseline to day 210 and LDL-C reductions were sustained over the window of 4 years without loss of efficacy, showing a significantly longer-lasting effect of inclisiran compared with that of the anti-PCSK9 mAb despite the similar relative reduction in LDL-C and PCSK9 levels. There were no new adverse event profiles during the 4-year study period. In addition, the ORION-14 (NCT04774003) and ORION-15 (NCT04666298) trials were conducted to assess the efficacy and safety of inclisiran in the Chinese and Japanese populations, respectively, and the ORION-18 trial (NCT04765657) is currently ongoing in Asian high-risk patients.

The ORION-10 (NCT03399370) and ORION-11 (NCT03400800) trials were double-blind, randomized, placebo-controlled, phase 3 trials to evaluate the percent change in LDL-C levels at the day 510 follow-up in 1561 patients with ASCVD in the United States and 1617 patients with ASCVD or risk equivalent in the European Union and South Africa, respectively [45]. The mean LDL-C levels at baseline in these two trials were 104.7 mg/dL and 105.5 mg/dL, respectively. At day 510, patients who received inclisiran achieved an LDL-C level reduction of 52.3% in the ORION-10 and 49.9% in the ORION-11 trial. Adverse events were not significantly different between the inclisiran and placebo groups in both trials.

The ORION-9 trial (NCT03397121) was conducted to assess the efficacy and safety of inclisiran in patients with heterozygous familial hypercholesterolemia (HeFH) [46]. The mean baseline LDL-C level was 153 mg/dL despite all patients receiving a maximally accepted dose of statin therapy with or without ezetimibe. At day 510, the mean percent change in LDL-C levels was a reduction of 39.7% in the inclisiran group vs. an increase of 8.2% in the placebo group. The substudy according to HeFH genotype revealed consistent reduction in LDL-C levels across all types of genetic defects in patients with HeFH. In the study summarizing the ORION-9, -10, and -11 trials for very high-risk patients, inclisiran reduced the levels of LDL-C, apolipoprotein B, and non-HDL-C by 51%, 42%, and 46%, respectively, which was associated with a 24% reduction in the rate of cardiovascular events in the subanalysis [47, 48]. In addition, a meta-analysis of five randomized controlled trials of inclisiran also demonstrated favorable effects on multiple lipid/lipoprotein parameters and an acceptable safety profile [49]. The ORION-8 trial (NCT03814187) is an ongoing open-label extension of the ORION-3, -9, -10, and -11 trials to assess the long-term effects and safety of inclisiran up to day 990. In the network meta-analysis of non-statin lipid-lowering therapies, PCSK9 mAbs reduced LDL-C by 64.7% (95% CI 67.4–62.0%), whereas inclisiran was found to reduce LDL-C by 50.2% (95% CI 55.0–45.4%). Inclisiran was expected to provide similar improvement in LDL-C levels though PCSK9 mAbs were more efficacious at reducing LDL-C than inclisiran [50]. The addition of inclisiran to maximally tolerated statins plus ezetimibe enables LDL-C reduction of more than 80% as well as PCSK9 mAbs [51, 52].

Based on the data from the phase 3 ORION-9, -10, and -11 trials that have confirmed the tolerability and efficacy of inclisiran in the long-term, inclisiran (Leqvio®, inclisiran 284 mg/1.5 mL solution for injection in a prefilled syringe) was approved by the European Medicines Agency in 2020 and by the United States Food and Drug Administration in 2021 for primary hypercholesterolemia or mixed dyslipidemia. It needs to be stored at room temperature of 20–25 °C (68–77°F) with allowable excursions between 15 and 30 °C (59–86°F) [35].

The ORION-4 trial (NCT03705234) is an ongoing double-blind, randomized, placebo-controlled phase 3 trial which has enrolled approximately 15,000 patients with established ASCVD in the United Kingdom and United States to assess the effect of inclisiran on the cardiovascular outcomes over a median follow-up duration of 5 years. The estimated primary completion date is December 2024. This result is expected worldwide, as it will provide valuable information on the clinical benefits of inclisiran in cardiovascular event prevention. Currently, the VICTORION program including a part of the ORION studies is underway to assess the benefit of inclisiran therapy on the life of high-risk patients (Table 1). The VICTORION-2PREVENT trial (NCT05030428) is a phase 3 cardiovascular outcome trial for patients with established ASCVD in multiple countries. This global study is designed to assess whether inclisiran reduces the risk of 3-point-major adverse cardiovascular events, defined as a composite of cardiovascular death, non-fatal myocardial infarction, and non-fatal ischemic stroke. There are also many other ongoing trials performed to promote the different use of inclisiran in various clinical settings, such as VICTORION-INCEPTION (NCT04873934), -INICIATE (NCT04929249), -REAL (NCT05399992), -DIFFERENCE (NCT05192941), and -SPIRIT (NCT04807400).

The phase 2 ORION-2 trial (NCT02963311) in adult patients with homozygous familial hypercholesterolemia (HoFH) was a proof-of-concept pilot study conducted to confirm the dose and regimen for the subsequent phase 3 ORION-5 trial (NCT03851705) [53]. As reductions in LDL-C and PCSK9 levels by 300 mg of inclisiran sodium were observed in patients with HoFH without adjustment of dosing or regimen, the ORION-5 trial, a double-blind, placebo-controlled, open-label, multicenter trial, was initiated with the standard dose and regimen to assess the long-term effects, tolerability, and safety of inclisiran. The results have not yet been published. Moreover, the ORION-13 (NCT04659863) and -16 (NCT04652726) trials, which were designed to assess the efficacy and safety of inclisiran in adolescents aged 12 to 17 years with HoFH and HeFH, respectively, are ongoing [54]. These trials may provide promising therapeutic options for adults and children with severe familial hypercholesterolemia in the current settings of limited availability of treatment.

### Adverse events

The available clinical trial data on the safety of inclisiran have shown that it is overall safe and well tolerated. In the ORION trials, adverse events occurred both in the placebo and treatment arms with a relatively similar frequency. There were no drug-related serious adverse events, and most reported adverse events were mild and moderate, including myalgia, cough, musculoskeletal pain, mild rash and hyperpigmentation, headache, musculoskeletal pain, nasopharyngitis, and dizziness. In the pooled safety analysis of the ORION-9, -10, and -11 trials that included 3655 patients, the safety findings were similar in the inclisiran and placebo groups for 18 months, but injection site reactions were more frequent in patients receiving inclisiran (5.0% vs. 0.7%) [48]. Additionally, the results of the ORION-3 trial showed well-tolerated safety profile consistent with other trials during 4 years of follow-up [44]. The network meta-analysis of PCSK9 inhibitors revealed that inclisiran was identified as the top ranked drug in association with less serious adverse events by the surface under the cumulative ranking curve, and there were no findings that LDL-C lowering therapies with PCSK9 mAbs and inclisiran were associated with new-onset diabetes and neurocognitive disorders [55].

In addition, abnormal hematological effects were not found in these studies, although, in a previous study, another gene-silencing approach of selective protein inhibition by targeting its mRNA was associated with thrombocytopenia [56]. Furthermore, in the prespecified analysis of the ORION-1 trial, there was no evidence of significant increase in the levels of inflammatory biomarkers, such as interleukin-6 or tumor necrosis factor-α, or relevant formation of antidrug antibodies leading to a possible loss of function as illustrated with the anti- PCSK9 mAb bococizumab [57]. However, these results were obtained through a relatively short-term follow-up; therefore, longer-term data are needed to fully evaluate the safety profile of inclisiran. Recent meta-analysis of genetic association studies demonstrated that the exposure to LDL-C lowering genetic variants was associated with a high risk of new-onset type 2 diabetes [58]. It may suggest the potential adverse metabolic effects of inclisiran.

## Challenges in current lipid-lowering therapies and perspectives of inclisiran therapy

### Variability in LDL-C levels

Statin therapy is the gold standard treatment worldwide for the management of dyslipidemia and prevention of ASCVD; however, there is an individual variation in LDL-C levels, even in clinical trials that enrolled patients with greater adherence to treatment than that encountered in routine clinical practice [59]. Contemporary registry data demonstrated that less than half of the patients with established ASCVD achieved the target LDL-C level of < 70 mg/dL with high-intensity statin therapy [11, 12]. The LDL-C-lowering response to statin therapy varies according to age, sex, race, baseline LDL-C levels, genetic diversity, and various metabolic factors. Although adherence is an important factor as a determinant, the variability in LDL-C levels has been reported to be an independent predictor of adverse cardiovascular events as well as blood pressure variations [60–62]. A meta-analysis of statin clinical trials demonstrated that the variability in LDL-C level percent reduction was wide, ranging from modest to significant reduction [59]. Therefore, we need to consider possible poor responses to statin treatment in individual patients.

Attainment of consistently lower LDL-C levels without major variation is essential to obtain the greatest benefit from LDL-C-lowering therapy. The addition of ezetimibe to statin therapy has been shown to be associated with a lesser variation in the LDL-C-lowering response compared to that with statin therapy alone [63]. Furthermore, anti-PCSK9 mAbs combined with statin therapy offered a more sustained LDL-C level reduction among a large majority of patients in a sub-analysis of the FOURIER trial [64]. The combination therapy with statin and non-statin LDL-C-lowering drugs is an effective way to overcome the heterogeneity in the response to statin therapy.

### Poor treatment adherence

The major causes for the variation in LDL-C levels have been shown to be not only insufficient responses to drugs, but poor adherence by patients and low use of intensive LDL-C-lowering therapy by physicians. More importantly, these factors lead to increased ASCVD risk and mortality rates in daily clinical settings [65–67]. Despite the substantial benefits from statin therapy, the adherence to the guideline-recommended lipid-lowering therapy with high-intensity statins is suboptimal in both patients and physicians. The Patient and Provider Assessment of Lipid Management (PALM) registry, a study performed to assess the current practice of lipid management in 5693 patients at outpatient clinics in the United States, observed that 25% of guideline-eligible patients failed to receive statins and 15% of clinicians continued to have concerns about the safety of statins despite their belief of benefit [66, 68]. In addition, the EUROASPIRE V study revealed that statin therapy was interrupted or its intensity was decreased in approximately 20% of patients because of statin intolerance or based on physician’s advice, according to the medical records and interviews [12].

The most common causes of statin intolerance have been shown to be statin-associated muscle symptoms; however, a meta-analysis of the results from randomized trials of statins revealed that more than 90% of muscle symptoms in patients receiving statin therapy were not due to the statins [69]. Typically, the average incidence of myopathy is approximately one case per 10,000 patients treated per year, and that of rhabdomyolysis is 2–3 cases per 100,000 patients [70, 71]. Moreover, a series of n-of-1 trials among statin-intolerant patients found no difference in the frequency of muscle symptoms compared with placebo, suggesting a nocebo effect among users of statins [72]. These results imply that the fear of adverse effects caused by confusing information regarding the side effects of statins could be a contributing factor in poor adherence.

Other potential contributors to poor adherence to lipid-lowering therapy include inconvenience of administration, high cost, low health literacy, poor awareness, and clinical inertia. In particular, physicians should avoid complex treatment regimens, frequent dosing, and increased number of pills to improve treatment adherence in daily practice [73, 74]. Adherence to dosing regimens remains challenging because oral small molecules require daily dosing and anti-PCSK9 mAbs are administered by subcutaneous injection once or twice a month via self-injection or with the help of a caregiver. Additionally, anti-PCSK9 mAbs are more expensive than other lipid-lowering therapies, which hampers treatment adherence despite the bi-weekly administration [75, 76]. A pooled analysis of the results from anti-PCSK9 mAbs clinical trials that included 4197 patients demonstrated a high level of adherence for over 1 year, whereas a cohort study including 13,151 patients in real-world clinical settings revealed that the anti-PCSK9 mAb therapy was discontinued within 6 months after treatment initiation in approximately 40% of patients [77, 78]. Moreover, in a survey to evaluate the patients’ experience with anti-PCSK9 mAbs, 33.7% of 1216 patients who initiated treatment discontinued it because of its high cost and lack of insurance approval [79].

### Expectations and challenges of inclisiran therapy

Inclisiran provided robust and sustained reduction in LDL-C levels in patients receiving the maximum tolerated dose of statins while achieving high consistency in individual LDL-C-lowering responses. In the ORION-1 trial, more than 95% of patients who received two doses of inclisiran of 300 mg experienced a persistent response that had not returned to within 20% of the change from the baseline LDL-C level at day 180 [80]. As a more than 50% reduction in LDL-C levels persists over 4 months after the second administration of inclisiran of 300 mg, the low inter-individual variability in LDL-C levels over time may provide a potential benefit to reduce the risk of cardiovascular events in a large number of high-risk patients. The degree of individual variation in the response to inclisiran is smaller than that for other LDL-C-lowering therapies, which could be due to the mechanism of direct inhibition of PCSK9 synthesis as opposed to the extracellular inhibition by anti-PCSK9 mAbs.

**Fig. 3:**
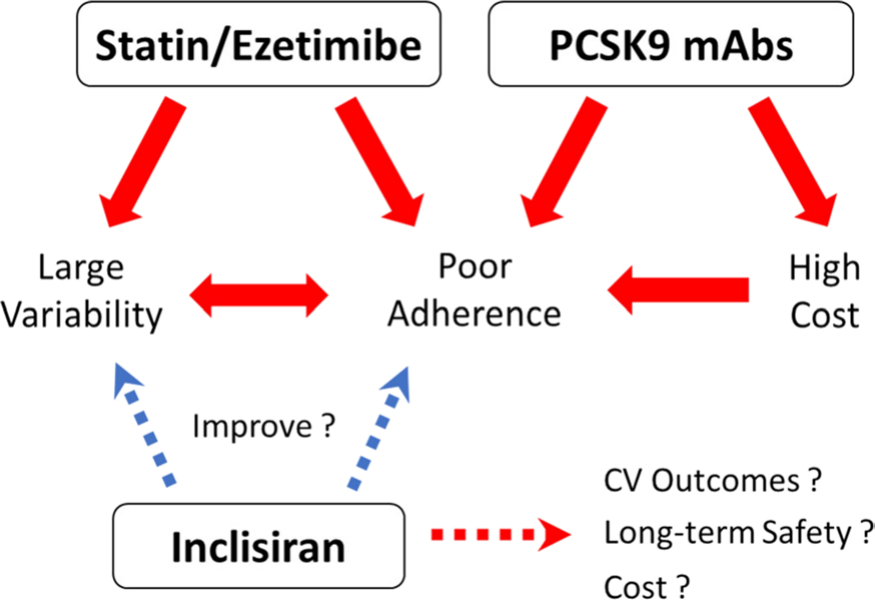
Potentials and challenges of inclisiran in current lipid lowing therapies. The individual variability in response to statins/ezetimibe and poor treatment adherence to daily administration remain challenging issues in primary care. PCSK9 monoclonal antibodies provide a consistent reduction in LDL-C levels, but poor adherence due to subcutaneous injection every 2- or 4-weeks and high cost are barrier. Robust and durable reduction in LDL-C with inclisiran has the potential to improve both large variability in LDL-C levels and poor treatment adherence, on the other hand, there are some challenges such as high cost, lack of cardiovascular outcome data, and longer-term safety over 5 years. *CV outcomes* cardiovascular outcomes, *PCSK9 mAbs* monoclonal antibodies

The greatest advantage of inclisiran is the infrequent, biannual administration schedule, which is expected to greatly improve treatment adherence despite its robust LDL-C-lowering effect being similar to that of anti-PCSK9 mAbs in combination with the maximal tolerated statin/ezetimibe therapy. Because the anti-PCSK9 mAbs bi-weekly dosing schedule by subcutaneous injection negatively impacts treatment adherence, the biannual dosing regimen of inclisiran may be considered in patients with poor adherence to anti-PCSK9 mAb therapy and those unable to self-inject anti-PCSK9 mAbs. Administration twice a year by a healthcare provider may completely solve the problem regarding treatment adherence. Additionally, as an annual injection of inclisiran results in a more than 30% reduction in LDL-C levels over a year, it would be possible to apply this regimen for primary prevention, for example, along with the flu vaccine (Fig. 3).

Nonetheless, a major barrier to the wide implementation of inclisiran is its cost-effectiveness and the lack of adequate data on cardiovascular outcomes and long-term safety. The cost of inclisiran will likely limit its widespread use in daily clinical practice. The Institute for Clinical and Economic Review (ICER) conducted a cost-effectiveness analysis and proposed a value-based price range of inclisiran reflecting the health economic outcome. This benchmark price range of inclisiran is USD 3600–6000 per year to be cost-effective, while the current average annual price for anti-PCSK9 mAbs is approximately USD 5400–5850 [81, 82]. However, the price of publicly available inclisiran was set to USD 3250 per dose, or USD 6500 annually based on the biannual two-dose regimen, which might result in high out-of-pocket costs for individual patients and increased financial burden to healthcare systems. To avoid limiting its use due to the high cost, as for anti-PCSK9 mAbs, the price needs to be lowered to a level that is affordable and acceptable for most high-risk patients.

In addition, there is no evidence on whether inclisiran provides greater cardiovascular benefits than those obtained with anti-PCSK9 mAbs. The results of the ORION-4 trial evaluating cardiovascular outcomes are eagerly awaited. However, meta-analyses of the results from the ORION-9, -10, and -11 trials showed that inclisiran was expected to reduce the rate of major adverse cardiovascular events by as much as 24–30% [47, 83]. In terms of the safety of inclisiran, the ORION trials reported a relatively favorable safety profile, but the duration of the follow-up period was relatively short. Therefore, the ongoing ORION-8 long-term extension study after the ORION-9, -10, and -11 trials will provide additional information on the safety of inclisiran for up to an additional 3 years. As the concern about statin safety based on widespread disinformation or confusion among both patients and physicians was associated with suboptimal statin use, the safety of inclisiran should be evaluated carefully and accurate information should be provided about its side effects. Furthermore, it is important that its long-term safety over decades is demonstrated, as it is the first-in-class siRNA therapeutic compound against PCSK9.

## Conclusions

Inclisiran is a novel siRNA molecule that inhibits PCSK9 synthesis, providing robust and sustained LDL-C level reduction with low inter-individual variability in the LDL-C-lowering response. The convenience of a biannual administration schedule is expected to greatly improve treatment adherence, which might contribute to better clinical outcomes in a large number of high-risk patients. However, lowering its price should be considered to promote its use in a broader range of patient populations for ASCVD prevention. Moreover, further study is needed to evaluate the long-term safety of this promising siRNA therapy.

## Data Availability

Not applicable.

## Abbreviations

ASCVD: Atherosclerotic cardiovascular disease
ASGPRs: Asialoglycoprotein receptors
GalNAc: Triantennary *N*-acetylgalactosamine
HeFH: Heterozygous familial hypercholesterolemia
HoFH: Homozygous familial hypercholesterolemia
LDL-C: Low-density lipoprotein cholesterol
LDLR: Low-density lipoprotein receptor
mAb: Monoclonal antibody
mRNA: Messenger ribonucleic acid
PCSK9: Proprotein convertase subtilisin/kexin type 9
siRNA: Small interfering ribonucleic acid
